# Preventable Society

**Published:** 1861-02

**Authors:** 


**PREVENTABLE SOCIETY.**—That philanthropic and able lecturer, Dr. E. Y. Robbins, says that a society was formed in England several years ago, under the patronage of the titled persons of the realm—Lords, Ladies, Reverends, Dukes, Duchesses, and others—the object of which was to prevent the children of the poor from getting sick. This is a wise, civic economy. A pair of good shoes, a thick woolen under-shirt, a few dollars expended in mending a leaky roof, or to supply an abundance of pure water to a household, would many a time be the means of warding off sickness from individuals and even whole families, otherwise doomed to weary years of invalidism, with the attendant expenses which have to be supplied from charitable funds.

"Doctor, I will give you all I possess, if you can save my last and only child," is not an unfrequent appeal in our office. In multitudes of such cases, one sentence of information, one moment's rational reflection, one hour's time, would have once averted a malady which now no human agency can alleviate, let alone cure. A blasted, blighted life, an age of remediless suffering, of untold agony, might be prevented in multitudes of cases, by impressing one single lesson on the subject of damp stockings, checked perspiration, cooling off too quickly after exercise, standing still a moment in a raw wind while in a heated condition from physical exertion, sleeping in damp sheets, resisting the calls of nature, going to bed immediately after a hearty supper, taking a bath immediately after dinner, sleeping in a small, close room. A well-learned lesson on any single one of these points would save many from wasting sickness and premature death. Does one parent in a dozen give one such lesson ?

